# Retinoic acid and cancer treatment

**DOI:** 10.7603/s40681-014-0022-1

**Published:** 2014-11-28

**Authors:** Mei-Chih Chen, Shih-Lan Hsu, Ho Lin, Tsung-Ying Yang

**Affiliations:** 1 Department of Medical Research, Taichung Veterans General Hospital, Taichung 407, Taichung, Taiwan; 2 Department of Life Sciences, National Chung Hsing University, No. 250, Kuokuang Rd., Taichung 402, Taichung, Taiwan; 3 Department of Chest Medicine, Taichung Veterans General Hospital, No. 160, Taichung Harbor Rd., Sec. 3, Taichung 407, Taichung, Taiwan

**Keywords:** Retinoic acid, Cancer

## Abstract

Retinoic acid which belongs to the retinoid class of chemical compounds is an important metabolite of vitamin A in diets. It is currently understood that retinoic acid plays important roles in cell development and differentiation as well as cancer treatment. Lung, prostate, breast, ovarian, bladder, oral, and skin cancers have been demonstrated to be suppressed by retinoic acid. Our results also show that low doses and high doses of retinoic acid may respectively cause cell cycle arrest and apoptosis of cancer cells. Also, the common cell cycle inhibiting protein, p27, and the new cell cycle regulator, Cdk5, are involved in retinoic acid’s effects. These results provide new evidence indicating that the molecular mechanisms of/in retinoic acid may control cancer cells’ fates. Since high doses of retinoic acid may lead to cytotoxicity, it is probably best utilized as a potential supplement in one’s daily diet to prevent or suppress cancer progression. In this review, we have collected numerous references demonstrating the findings of retinoic acid in melanoma, hepatoma, lung cancer, breast cancer, and prostate cancer. We hope these observations will shed light on the future investigation of retinoic acid in cancer prevention and therapy.

## 1. Introduction

Vitamins are nutrients essential for the body’s growth, differentiation, development, and protection., Vitamin A is especially important because it can’t be synthesized by animals and must be supplied from a diet that includes plants [1]. There are many derivatives of vitamin A, including β-carotene, retinol, retinal, isotetrinoin, and retinoic acid. Treatment using retinoic acid was approved by the U.S. Food and Drug Administration for lymphoma [2] and leukemia [3]. Since retinoic acid is known to be effective in treating cancer, its basic structure has been well identified. All of the retinoids, including retinoic acid, are comprised of three units: a bulky hydrophobic region, a linker region, and a polar region (carboxylic acid terminus). There are many compounds derived from the above basic structure, and these compounds are collectively called retinoids [1]. Due to the efficiency of natural retinoids in cancer treatment, synthetic retinoids have been generated and investigated. In anti-cancer research, retinoic acid has been investigated and found to inhibit the markers of cell proliferation, such as cyclin D1 and human telomerase reverse transcriptase (hTERT), and growth factor, such as epidermal growth factor receptor (EGFR) and vascular endothelial growth factor (VEGF) [1]. The biological functions inhibited by retinoic acid include tumor growth, angiogenesis, and metastasis [1]. In addition, retinoic acid has also been found to regulate mitochondrial permeability, death receptors, ubiquitination, and reactive oxygen species, etc. [4]. It is believed that the inhibitory effects of retinoic acid are achieved through activating the retinoic acid receptor (RAR) or retinoic X receptor (RXR). RAR and RXR form heterodimers and function after ligand binding. To turn on downstream gene expression, RAR and RXR shuttle into cell nuclei and bind to the retinoic acid response elements (RARE), which are located in the 5’-region of retinoic acid downstream genes [5]. The activation of the above classical pathway will lead to cell differentiation, arrest, and eventually apoptosis [6]. In addition to the above classic pathway, retinoic acid may also regulate the downstream gene expression through modulating other transcription factors, such as NF-κB, IFN-γ, TGF-β, MAPK, and even chromatin remodeling [4]. RARs/RXRs heterodimerize with other receptors and regulate these partner receptors’ signaling, including non-classical or non-genomic pathways [7]. Sometimes, these partner receptors have opposite functions to RARs/RXRs. The latest finding of retinoic acid is the regulation of stem cell differentiation [8]. Ying *et al.* found that retinoic acid induces the expression of lineage-specific differentiation markers Tujl and GFAP and reduces the expression of neural stem cell markers such as CD133,

Msi-1, nestin, and Sox-2 [8]. In their expression microarray analysis, retinoic acid-affected pathways include retinoid signaling and metabolism, cell adhesion, cell-matrix interaction and cytoskeleton remodeling. Notch pathway down-regulation was also reported by retinoic acid-induced HES and HEY inhibition [8].

Although there are several lines of evidence indicating the effects and mechanisms of retinoic acid in cancer therapy, the chemo-prevention and therapeutic application of retinoic acid remain controversial. Here, this mini-review article demonstrates an overview of the research to date in the field of retinoic acid application and therapy to various types of cancer. The hope is that this review may impart readers with a better understanding of the research history of retinoic acid as well as guide the future direction in the field.

## 2. Retinoic acid and melanoma

Retinoic acid has been found to have inhibitory effects on growth of murine melanomas [9] and colony formation of human melanomas [10]. Activations of cyclic AMP-dependent protein kinase and sialyltransferase have also been found to involve the effects of retinoic acid [7, 11]. On the other hand, the modulation of melanoma cell adhesion to basement membrane components has been shown to be affected by retinoic acid treatment [12, 13]. Intercellular adhesion molecule gene I (ICAM-1) is transcriptionally regulated by retinoic acid in melanoma cells [14]. Retinoic acid has also been indicated to inhibit highly metastatic B16F10 melanoma cells by down-regulating the cell surface integrin receptors against extracellular matrix proteins, specifically laminin and vitronectin [15]. Since the formation of melanoma is correlated to radiation, retinoic acid has been found to modify the radio-sensitivity and recovery from X-ray damage *in vitro* [16]. Notably, the induction of protein kinase C in mouse melanoma cells was identified by retinoic acid treatment [17]. Ultraviolet irradiation may deplete cellular retinol and alter the metabolism of retinoic acid in cultured human keratinocytes and melanocytes [18]. In addition to inhibiting growth, retinoic acid has been found to inhibit human melanoma tumor cell invasion [19]. Epidermal growth factor receptor (EGFR) is a crucial player in epithelial cells in both growth and migration/invasion. Yongshan *et al.* discovered that EGFR expression was regulated by retinoic acid treatment [20]. In 1993, the combination treatment of interferon-α and retinoic acid was first believed to have significant therapeutic effect on melanoma by clinical examination [21]. The antitumor effect of green tea polyphenol on melanoma was enhanced by retinoic acid [22]. Interestingly, the differential regulation of tyrosinase activity in the skin of white and black individuals *in vivo* by retinoic acid was demonstrated [23]. In regards to drug delivery improvement, retinoic acid was encapsulated by liposome to treat melanoma cells and was then implanted onto C57BL/6 mice, with result of metastatic ability being efficiently suppressed [24]. A hyaluronic acid-based multifunctional nano-carrier was also used to deliver retinoic acid in cancer treatment tests [25]. All things considered, Retinoic acid seems to be a promising treatment for melanoma and more details will be investigated in the future to strengthen the basis of its mechanism.

## 3. Retinoic acid and hepatoma

Hepatoma is a serious form of cancer in Asia. It has been found that retinoic acid may directly cause the increase in protein synthesis of transferrin and albumin in Hep3B cells [26]. Since hepatitis virus infection is important to hepatoma formation, Hsu *et al.* demonstrate that retinoic acid may regulate the gene expression of hepatitis B virus surface antigen (HBsAg) in hepatoma cells [27]. Much cancer research focuses on the involvement of topoisomerase in cancer cell growth. Tsao *et al.* has reported that retinoic acid represses the expression of topoisomerase II in Hep3B cells [28]. The most current research of retinoic acid has used the model of short-term treatment and therefore been questioned in clinical therapy. However, Hsu *et al.* have demonstrated that long-term treatment with retinoic acid (30 days) may lead to suppression of the tumorigenicity of human hepatoma cells [29]. Furthermore, apoptosis of hepatoma cells was found after retinoic acid treatment and prevented by serum albumin and enhanced by lipoidol [30]. In addition, p21 induction and cdc2 activation are found to involve retinoic acid-induced hepatoma apoptosis [31]. Since retinoic acid may cause detachment of cancer cells under serum starvation, proteolysis of integrin α5 and β1 subunits were found in hepatoma cells [32]. The latest research indicates that retinoic acid may cooperate with arsenic to induce apoptosis and modulate the intracellular concentration of calcium in hepatoma cells [33]. Additionally, the retinoic acid receptor-related receptor α is believed to be a prognostic marker for hepatoma [34]. Taken together, these observations elucidate the fact that retinoic acid is indeed a potential compound to suppress hepatoma growth and cause hepatoma apoptosis. It’s also possible that retinoic acid can work as a helper that cooperates with other treatments and attacks hepatoma.

## 4. Retinoic acid and lung cancer

The incidence and mortality rates of lung cancer make this disease an important topic in cancer research. Since the relevant contribution of retinoic acid in cancers was discovered, there have been numerous studies demonstrating the effects of retinoic acid in lung cancer progression. At first, Hsu *et al.* found retinoic acid-mediated G1 arrest to be associated with induction of p27 and Cdk3 inhibition in lung squamous carcinoma cells [35]. In C57BL/6 mice model, retinoic acid was encapsulated and inhibited lung cancer metastasis [36]. Syndecan-1 is a proteoglycan that mediates cell-cell adhesion and prevents invasion in epithelial cells. Retinoic acid may increase syndecan-1 expression to block invasion/metastasis of lung cancer [37]. Notably, retinoic acid has been found to reduce chemotherapy-induced neuropathy in an animal model as well as patients with lung cancer [38]. These results show the relevance of retinoic acid in lung cancer treatment.

## 5. Retinoic acid and breast cancer

The application of retinoic acid in breast cancer treatment was first mentioned in 1970’s [39]. A retinoic acid-binding protein is believed to be an important factor in the progression of breast cancer [40, 41]. The latest report indicates that the sensitivity of retinoic acid in triple negative breast cancer cell lines may be restored by other treatment, such as curcumin [42]. Aldehyde dehydrogenase 1A3 (ALDH1A3) influences breast cancer progression *via* differential retinoic acid signaling [43]. Besides the above, a different type of protein kinase C was also found to involve the induction of the retinoic acid system in breast cancer [44]. Notably, retinoic acid may induce re-differentiation of early transformed breast epithelial cells [45], suggesting the preventive role retinoic acid plays with respect to breast cancer. Kamal *et al.* drew attention to the effect of retinoic acid by proteomic analysis in breast cancer cell lines [46]. The amplification of the retinoic acid receptor α (RARα) and retinoic acid sensitivity were found to correlate to breast cancer progression [47]. Retinoic acid can impair estrogen signaling in breast cancer cells by interfering with the activation of LSD1 via protein kinase A [48]. Retinoic acid was also found to reduce breast cancer growth and lung metastasis [49]. The procoagulant activity of breast cancer cells was reported to be modulated by retinoic acid [50]. Interestingly, microRNA-21 was found to be induced by retinoic acid in breast cancer, which suggests the biological correlation and molecular targets in breast cancer [51]. In addition, retinoic acid may inhibit aromatase activation and expression, which indicates that the estrogen supply inside breast cancer cells is insufficient to maintain cancer cell growth [52]. In addition to growth inhibition, retinoic acid is able to down-regulate MMP-9 by modulating its regulatory molecules and therefore impacts the invasion ability of breast cancer cells [53]. Additionally, retinoic acid may inhibit telomerase activation through inducing histone deacetylation in estrogen receptor-negative breast cancer cells [54]. Importantly, Hau *et al.* elucidated the genomic antagonism between retinoic acid and estrogen signaling in breast cancer and published their findings in the journal, Cell [55]. Their article shows the critical and solid thought of retinoic acid application to breast cancer. Since HOXA5 plays a role in apoptosis of breast cancer cells, retinoic acid was reported to regulate HOXA5 through RAR-β [56]. Cell cycle control gene, Btg2, is believed to be a direct target for RAR signaling in breast cancer cells [57]. Moreover, retinoic acid may sensitize breast cancer cells to taxol through down-regulation of survivin and promote the aberrant mitotic progression that causes apoptosis [58]. Although a lot of evidence demonstrates the effectiveness of the application of retinoic acid to breast cancer, combination treatments with other effective compounds (such as tomaxifen, taxol, and interferone) has been proposed and is currently utilized.

## 6. Retinoic acid and prostate cancer

Just like breast cancer, the history of retinoic acid treatment for prostate cancer has a strong history going back to the 1980’s. Researchers’ attention then was focused on the retinoic acid receptor in the study of prostate cancer cells [59]. The effects of retinoic acid on the growth and morphology of a prostate cancer cell line was first investigated [60]. After that, the binding proteins of retinoic acid were identified [61-63]. Since prostate cancer cells are eager to require androgen supplement in the early stages of the disease, 5α-reductase becomes important to provide potent androgens. Retinoic acid was found to inhibit 5α-reductase and therefore became a possible treatment for prostate cancer [64, 65]. Notably, the relationship between retinoic acid and prostate cancer growth was officially mentioned by Whelan [66]. Fong *et al.* demonstrated that retinoic acid at 10 μM may cause inhibition of androgen-dependent prostate cancer cell growth but may cause stimulation when the concentration is 0.01 μM [67]. The growth of androgen-independent prostate cancer cells is also suppressed by retinoic acid [68]. Extracellular matrices were also found to be regulated by retinoic acid [69, 70]. Specifically, retinoic acid has been found to activate the tumor suppressor, Rb, and decline androgen receptor proteins, thereby causing apoptosis of prostate cancer cells [71]. Interestingly, retinoic acid has been reported to interact with androgen in prostate cancer cells, which affects cell proliferation and expressions of retinoic acid receptor and epidermal growth factor receptor [72]. There is some research that demonstrates that the retinoid X receptor (RXR) might play important roles in tumorigenesis of prostate [73, 74]. RXR was also found to involve retinoic acid-induced inhibition of androgen receptor [75]. Hypermethylation of the retinoid acid receptor β is believed to be a prognostic marker in prostate cancer [76, 77]. Notably, the retinoic acid synthesis gene aldehyde dehydrogenase, ALDH1A2, is believed to be a candidate tumor suppressor in prostate cancer [78], which is similar to breast cancer as described above. More solid evidence has been provided by Huss *et al.,* in which they have indicated that retinoic acid may slow the progression of prostate cancer and promote apoptosis of cancer cells [79]. In addition, retinoic acid was found to regulate the formation and degradation of gap junctions in prostate cancer cells [80]. Also, retinoic acid may inhibit the proliferation of prostate cancer cells through reducing the methylation level of the HOXB13 gene [81] and the Cdk5-dependent p27 expression [82]. Instead of growth inhibition, high doses of retinoic acid may cause apoptosis of prostate cancer cells though p35 cleavage and Cdk5 overactivation [83]. Although clinical trials have not shown strong evidence indicating that retinoic acid is an effective drug for prostate cancer [84, 85], more and more effort has been put toward retinoic acid research as it relates the nutritional supply and combination therapies with respect to prostate cancer.

## 7. Conclusion

Retinoic acid has been investigated extensively for its use in treating different forms of cancer not only in prevention but also in treatment. In this review, we described the research and applications of retinoic acid in melanoma, hepatoma, lung cancer, breast cancer, and prostate cancer. As a nutrient, retinoic acid may be obtained from either through the daily metabolization of plants in a balanced diet or through vitamin supplements. Under normal circumstances in the body, retinoic acid does preventive work against cancer formation. After cancer formation, retinoic acid becomes an attacker to cancer cells, one that blocks their growth and division and also triggers their differentiation and death through specific pathways. Furthermore, retinoic acid has been proven to cooperate with other effective cancer therapeutic drugs against cancer progression. Retinoic acid becomes a helper to chemo-therapeutic agents, a helper which may decrease both the dosages of these chemo-therapeutic agents required and their side-effects. This may relieve patients’ pain from chemotherapy and improve patients’ quality of life. From these points of view, although there has been a long history and no small amount of controversy regarding retinoic acid application in cancer treatment, it’s still worthwhile to continue research and place future effort toward gaining a more complete understanding of the application of retinoic acid in cancer treatment.

## Acknowledgements

The study was supported by Taiwan Ministry of Science and Technology with regular grant (NSC 101-2320-B-005-004-MY3 to H.L.) and 2014 New Partnership Program for the Connection to the Top Labs in the World (103-2911-I-005-507/104-2911-I-005-501 to H.L.).
